# A Novel RGB-D SLAM Algorithm Based on Cloud Robotics

**DOI:** 10.3390/s19235288

**Published:** 2019-12-01

**Authors:** Yanli Liu, Heng Zhang, Chao Huang

**Affiliations:** School of Information Engineering, East China Jiaotong University, Nanchang 330013, China; 2017068085211011@ecjtu.edu.cn

**Keywords:** RGB-D SLAM, cloud robotics, ROS, 3D point cloud

## Abstract

In this paper, we present a novel red-green-blue-depth simultaneous localization and mapping (RGB-D SLAM) algorithm based on cloud robotics, which combines RGB-D SLAM with the cloud robot and offloads the back-end process of the RGB-D SLAM algorithm to the cloud. This paper analyzes the front and back parts of the original RGB-D SLAM algorithm and improves the algorithm from three aspects: feature extraction, point cloud registration, and pose optimization. Experiments show the superiority of the improved algorithm. In addition, taking advantage of the cloud robotics, the RGB-D SLAM algorithm is combined with the cloud robot and the back-end part of the computationally intensive algorithm is offloaded to the cloud. Experimental validation is provided, which compares the cloud robotic-based RGB-D SLAM algorithm with the local RGB-D SLAM algorithm. The results of the experiments demonstrate the superiority of our framework. The combination of cloud robotics and RGB-D SLAM can not only improve the efficiency of SLAM but also reduce the robot’s price and size.

## 1. Introduction

Simultaneous localization and mapping (SLAM) is an important research topic in the field of autonomous mobile robots, and it is also the key for mobile robots to achieve autonomous navigation and perform tasks in an unknown environment, which embodies the robot’s perception ability and intelligence level. In recent years, the theories and methods of 2D map building have been comprehensively studied and fruitful results have been achieved. With the progress of sensor technology and the continuous development of SLAM computing theory, the 3D map building of a 6-DOF (degree of freedom) robot has attracted the attention of researchers.

For SLAM research, sensors used in the early stage include laser radar and cameras. Although the laser radar can accurately capture the 3D information of objects and has good robustness, it is more expensive and requires IMU (inertial measurement unit) [1] sensors for SLAM. Kinect is an RGB-D (R(red), G (green), B (blue), D (depth)) sensor, which is officially released by Microsoft in June 2010. As Kinect can capture the depth image and RGB image at the same time, the frame rate is high and the price is relatively low. With its excellent performance, Kinect has been favored by researchers in the field of robotics and achieved unexpected results. In recent two years, the depth-sensing technology of Kinect has been extended. Compared to stereo cameras and time-of-flight cameras, Kinect has many advantages [2,3], such as low price, information integrity, and complex environmental adaptation.

Compared with the traditional SLAM technology, RGB-D SLAM has better real-time performance and less computation for data analysis than traditional SLAM, which is more suitable for the construction of a 3D environment map. However, there are still many problems to be solved to realize the high quality and efficient map construction process by using RGB-D SLAM [4,5,6,7]. For example, when the robot moves from a narrow passage to an open room, the environment changes a lot, and the depth information feedback is easily distorted. Map building and map fusion tasks in SLAM often consume a lot of computing resources. With the expansion of maps, more and more features need to be retrieved. If the processing is not timely enough, the robot may deviate from the original motion track at any time.

3D map construction needs to store and calculate a large amount of image information, and the hardware configuration of the local computer is relatively high. The medium and low configuration robot cannot meet the requirements of large data storage and intensive calculation in the 3D map construction process. The emergence of cloud robot technology [8,9,10] provides a new way of thinking about this problem. Local computers do not need to calculate and store the 3D map in the 3D map construction based on cloud robot technology, but only need to unload the intensive computing and mass storage to the cloud platform.

In this paper, the original RGB-D SLAM algorithm [11] is briefly described, and the shortcomings of the algorithm are analyzed. To solve the disadvantages of the original RGB-D SLAM, such as low efficiency and poor real-time performance, we have improved several key links of the RGB-D SLAM algorithm. Cloud robot technology provides a new direction for solving the problems of the RGB-D SLAM algorithm, such as intensive computing and huge data. This paper combines these two technologies to provide a new solution for improving the efficiency and real-time performance of the RGB-D SLAM algorithm. The main contributions of this paper are as follows: (1) We improve 3D point cloud registration based on the SVD (singular value decomposition) algorithm, which is used for the cloud map fusion. (2) We improve the RGB-D SLAM algorithm from three aspects: feature extraction, multi-scene point cloud stitching, and the optimization of the pose graph. (3) We present a novel RGB-D SLAM algorithm based on cloud robotics, which offloads intensive computational tasks to the cloud.

For (2), RGB-D SLAM algorithm is improved from three aspects. (a) Feature extraction is used by the BRISK _D algorithm [12], which efficiently combines FAST (features from accelerated segment test) and BRISK (binary robust invariant scalable keypoints) methods. (b) Multi-scene point cloud stitching based on SVD algorithm and SVD (singular value decomposition) algorithm is used to solve the transformation matrix of point cloud registration. (c) Pose optimization with HOG-Man algorithm (hierarchical optimization for pose graphs on manifolds).

The rest of the paper is organized as follows. Section 2 refers to the related work. Then the original RGB-D SLAM algorithm is described in Section 3. In Section 4, the improvement of the RGB-D SLAM algorithm is introduced. The RGB-D SLAM algorithm with the cloud robot is presented in Section 5. Some experimental results are shown in Section 6, and Section 7 sets out the conclusions and future work.

## 2. Related Work

### 2.1. RGB-D SLAM

Henry et al. proposed the RGB-D SLAM method for the first time in 2010 [11]. This method uses both color and depth images. Firstly, SIFT (scale-invariant feature transform) [13] and random sample consensus (RANSAC) algorithm [14] are used to match image features. Then, ICP(iterative closest point) algorithm [15] is used to align images and TORO (Tree-based netwORk Optimizer) algorithm [16] is used to optimize the processing, and finally, the 3D environment map is constructed.

Henry et al. further optimized the RGB-D SLAM method in 2012 [17]. This method uses FAST (features from accelerated segment test) corner detection [18] and Calonder feature descriptor [19] instead of the SIFT algorithm, and uses an inter-frame RANSAC algorithm for image feature registration. At the same time, SBA (sparse bundle adjustment) algorithm [20] is used for map optimization processing, and finally, a 3D environment map is constructed.

Sturm et al. [21] present a method for evaluating the position accuracy of RGB-D SLAM system in 2012, and disclose the data set they used, which includes color and depth images captured by RGB-D cameras in two different rooms, and the real motion trajectory of RGB-D cameras captured by a high-precision motion capture system. At the same time, this paper also provides a method to calculate the position accuracy of the RGB-D SLAM system. Researchers of RGB-D SLAM can use this evaluation method to test the performance of their system. This evaluation method greatly promotes the research work of RGB-D SLAM.

Endres et al. propose an RGB-D SLAM method in 2012 [22]. This method divides the RGB-D SLAM system into two parts: front-end and back-end. The front-end is responsible for establishing the spatial transformation relationship by processing sensor information, and the back-end is responsible for reducing cumulative error by graph optimization. This method uses SIFT, SURF (speeded up robust features) [23], and ORB (ORiented brief) algorithms [24] to extract features, and the RANSAC algorithm to process image alignment. Unlike Henry, the ICP algorithm is not used for image alignment, and then the g${}^{2}$o (general graph optimization) algorithm [25] is used for graph optimization. Finally, the method uses the Octomap library [26] to raster the final output point cloud map to form a grid map, which is convenient for robot navigation and localization. In addition, Endres et al. also use the location accuracy evaluation method by Sturm [21] to verify the proposed RGB-D SLAM method.

Scherer et al. propose an RGB-D SLAM method in 2013 [27]. This method uses the HOG-MAN algorithm [28] to optimize the graph in the back end of RGB-D SLAM, and finally construct a 3D environment map. The RGB-D SLAM method proposed by Scherer et al. in 2013 does not splice each frame of the image. Instead, it uses the Harris scoring function constructed by the number of FAST corner points as the criterion to filter the key frames, and then splicing these key frames. This method uses the BA (bundle adjustment) algorithm combined with the depth information to perform image registration on key frames.

Quang et al. make some improvements to the RGB-D SLAM method in 2015 [29]. This method only uses the RANSAC algorithm to establish the spatial transformation relationship, and then selects the key frames for image mosaic according to the magnitude of the transformation amplitude, instead of splicing each image, so as to reduce the cumulative error and improve the position accuracy of RGB-D SLAM system.

In 2011, Neumann et al. [30] use a GPU (graphics processing unit) to improve the processing efficiency of the RGB-D SLAM system. This method puts the process of processing point cloud data using the ICP algorithm on the GPU unit, and the experimental results prove the processing on the GPU unit. The time is less than 20 ms, which can greatly reduce the computational cost of the RGB-D SLAM system.

Lee et al. [31] propose a real-time RGB-D SLAM method based on GPU unit in 2012. This method uses the GPU unit to realize the parallelization of feature extraction and pose optimization to improve computational efficiency. In 2015, to solve the problem that it is difficult to match visual features in the simple steps by using visual sensors alone, Bmnetto et al. [32] construct a system combining vision and inertial measurement in 2015 and validate the effectiveness of this method by experiments. Aiming at the problem of less texture and ambiguous environment, Qayyum et al. [33] propose an RGB-D SLAM method combining the inertial navigation information in 2017, which can effectively reduce the error of pose estimation.

In 2018, Zhang et al. [34] propose a surface closed-loop visual SLAM framework (LoopSmart). It combines sparse feature matching and dense surface alignment strategies. Sparse feature matching is used for visual odometers and for camera global pose fine-tuning when dense loops are detected, while dense alignment is the method of large loops and solving surface mismatch problems. The framework is based on the tracking module of ORB-SLAM2 [35] and the intensive model fusion strategy is based on the surface model. They have performed a lot of experimental verification on different data sets and have a good effect on camera trajectory and 3D reconstruction accuracy.

In 2018, Ylimäki et al. [36] propose a method to deal with outliers even in the case of very serious registration error, aiming at the problem that the depth images of the RGB-D sensor will produce noise and lead to poor initial registration. This method is robust to abnormal and wrong depth measurements, as well as significant depth map registration errors caused by an inaccurate initial camera pose.

For dense RGB-D SLAM, previous work has focused on BA(Bundle adjustment) approximations. In 2019, Schöps et al. [37] present a novel RGB-D SLAM method with a real-time direct BA back-end, which allows one to use rich information during global optimization, resulting in very accurate trajectories.

After recent years of development, RGB-D SLAM technology tends to be mature [38,39] and has achieved good experimental results. However, the RGB-D SLAM algorithm still has some shortcomings. The advantages and disadvantages of the RGB-D SLAM algorithm are listed in Table 1.

In view of the two disadvantages of (1) and (2), we propose the improvement of the original RGB-D SLAM in Section 4. For the third disadvantage, in the construction of a large-scale scene map, with the continuous operation of robots in an unknown environment, more and more data are collected by the RGB-D SLAM system, and the amount of information that needs to be processed by its back end will increase correspondingly. Especially in the later stages of reconstructing 3D maps of larger scenes in RGB-D SLAM system, the amount of information needed to be processed in the back end will be doubled, seriously affecting the real-time performance of the system. So, the algorithm in this paper unloads the back-end computation to the cloud in the 3D reconstruction system for improving the real-time performance of the system.

### 2.2. “Cloud+Robot” SLAM

The development and current status of robot SLAM are described above. The introduction of cloud computing is of great significance to SLAM. On the one hand, the massive computing storage resources of the cloud itself are sufficient to deal with most SLAM algorithms. On the other hand, placing the SLAM module in the cloud instead of the robot side can greatly alleviate the resource burden of the robot and eliminate the cumbersome and expensive deployment work. In the combination of cloud robot technology and SLAM, many researchers have also proposed a number of different frameworks, such as DAvinCi [40], Rapyuta [41], and C${}^{2}$TAM [42].

#### 2.2.1. DAvinCi

DAvinCi [40] is one of the earliest software frameworks to enhance the capabilities of robots using cloud robotics. It aims to enhance the capability of robots in large and complex environments by utilizing the powerful computing and parallel processing capabilities of the cloud. In the actual demonstration, DAvinCi uses FastSLAM in SLAM to verify the feasibility of this architecture in paper [40], which proves that the cloud + robot can improve the performance of the robot, and it also proves that the computational storage resources required by SLAM are non-negligible or even unbearable burdens for the robot.

The DAvinCi framework [43] has some limitations. First of all, the robot in its architecture is passively using the cloud to improve performance rather than actively using the cloud according to its needs. It is necessary to set the scene and action of its use in the cloud beforehand, which is obviously impossible for the task of robots in large and complex environments. So, it determines that the DAvinCi framework cannot meet the current needs of robots. Secondly, its cloud architecture Hadoop has good performance in parallel processing tasks and computing but it has no obvious effect on tasks that cannot be processed in parallel. For SLAM, it is difficult to adapt to the different SLAM algorithms.

#### 2.2.2. Rapyuta

Rapyuta is the “brain” part of the European RoboEarth Project, which aims to improve robotic capabilities through a series of advantages of the cloud. Its core technology is to use LXC (Linux container) technology to build a virtual machine corresponding to a robot in the cloud. In paper [44], the SLAM experiment is performed using Rapyuta to verify the support and enhancement of the cloud for the low-cost robot SLAM under the Rapyuta framework.

Rapyuta’s main advantage [45] is that, first of all, its architecture is a typical PaaS (platform as a service) architecture, which provides the cloud function as a service to the robot. Robots have full rights of autonomous mobilization and use. Secondly, Rapyuta ensures strong support for robots with different shapes, hardware, and systems, and conforms to the characteristics of a unified platform.

Rapyuta has some limitations. Considering the task requirements of current robots, the tasks faced by robots are not single functional requirements, but a series of complex functions. Therefore, it is often necessary to run multiple different functional nodes on a virtual machine corresponding to a robot. At the same time, there is a wide gap between the resources and resource categories required by these functional nodes, which makes it necessary to consider the needs of different robots when building virtual machines for robots and change according to the different tasks of robots. Therefore, for the cloud, the robot-based computing unit is not conducive to cloud resource management.

#### 2.2.3. C${}^{2}$TAM

C${}^{2}$TAM is a “cloud + robot” framework for collaborative mapping, which adapts to the cooperative SLAM problem [46,47]. It aims to solve the problems of map operation, map reuse, map expansion, and multi-machine collaborative mapping in SLAM through the resource advantages of the cloud. C${}^{2}$TAM is based on PTAM (parallel tracking and mapping) method [48]. PTAM is based on the classical SLAM algorithm. According to the characteristics of the 3D map, the two processes of tracking and mapping are separated into two parallel steps from each iteration. On the one hand, the mapping side extracts common feature points through the observed images to form a whole image. On the other hand, the observer obtains the current position information by matching the current image with the whole image. C${}^{2}$TAM chooses to put the composition part of PTAM into the cloud because it consumes more resources. Thus, the efficiency of SLAM is improved.

Based on the SLAM algorithm in the cloud, C${}^{2}$TAM utilizes the separation of the PTAM mapping process and robot localization. On the basis of single machine SLAM, multi-machine collaborative mapping can be constructed by map fusion in the cloud. At the same time, the storage resources in the cloud are used to store the maps that have been constructed. When there are new mapping tasks, the automatic search and matching will be carried out to minimize redundant tasks. This process is called map reuse. If the machine acquires information that does not match the original map or information that is not included in the original map on the basis of matching the existing map, the cloud will update the map according to the new observation information. This process is called map extension.

The advantage of C${}^{2}$TAM architecture is that it makes full use of the cloud, and uses the PTAM method of 3D mapping to propose a more successful “cloud + robot” architecture and the corresponding processing methods are provided for various requirements in the SLAM process. In the paper [42], a detailed experimental verification of its various functions is carried out, which proves the availability and efficiency of its architecture. The ideas and methods of dealing with various SLAM scenario functions are worth reading for reference.

Its limitation [49] is that only one method of 3D mapping is proposed to solve the cloud problem. Robot SLAM has different optimal methods and algorithms according to its different service function scenarios. At the same time, the map’s needs will vary with the environment and people. C${}^{2}$TAM is obviously difficult to provide a general solution to the SLAM problem cloud.

#### 2.2.4. Comparison of Different Platforms

Each of these frameworks has its own advantages and disadvantages, as shown in Table 2.

There are connections and differences in working principles among different cloud architectures. Each framework used a different set of protocols that allow robots to offload the computation to the cloud. The Rapyuta framework is a more flexible framework than the other. Due to the open-source implementation of the Rapyuta framework, it can be extended to other robot functionalities easily. The DAvinCi framework has more advantages in parallelism, while the C${}^{2}$TAM framework is more suitable for large data storage and low bandwidth. As this paper discusses the SLAM issue, C${}^{2}$TAM framework is chosen as the cloud platform.

## 3. The Overall Algorithm Flow of Original RGB-D SLAM

With the development of Microsoft Kinect, a more innovative SLAM method emerges, that is, the RGB-D SLAM algorithm. In this section, we will introduce the original RGB-D SLAM algorithm [11] and analyze the shortcomings.

### 3.1. The Overall Algorithm Flow of RGB-D SLAM

**Figure 1:**
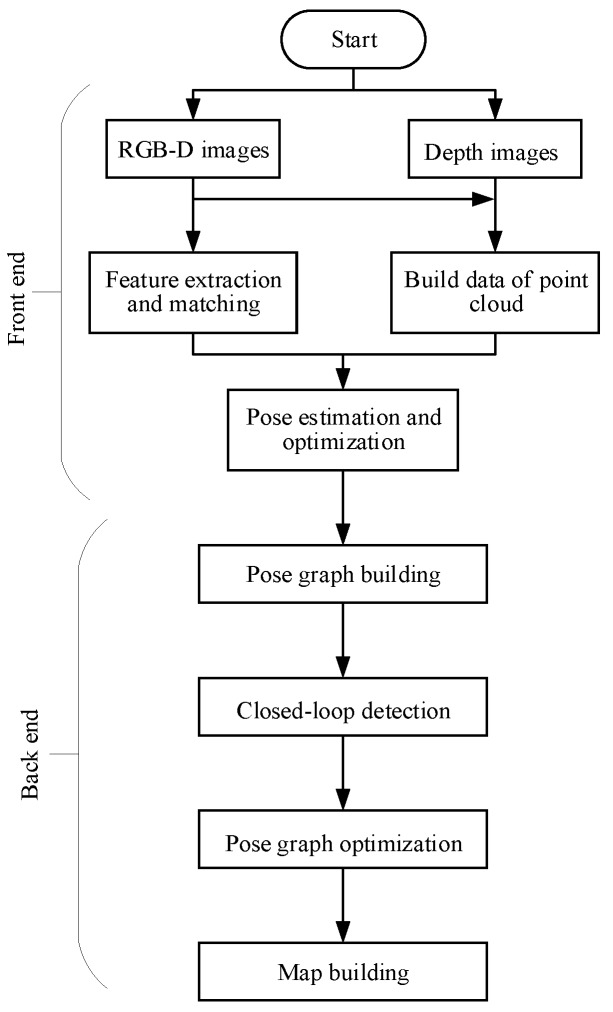
RGB-D SLAM algorithm flow.

The algorithm can be divided into two parts, the front-end and the back-end. The task of the front-end is to extract the spatial relationship between different observations. The task of the back-end is to optimize the pose information of the camera in the pose graph using a non-linear error function. The overall flow of its algorithm is shown in Figure 1.

In the front-end of the RGB-D SLAM algorithm, feature points detection and descriptor extraction are performed on each RGB image input by the camera, and feature points descriptors of two adjacent frames are matched. The depth information of these matching feature points is obtained from the corresponding depth image, and a set of 3D point correspondences between two adjacent frames is obtained. The 6D motion transformation is estimated according to the corresponding relation, and then the motion transformation is optimized to get the optimized 6D motion transformation.

In the back end of the RGB-D SLAM algorithm, a graph-based pose optimization method is applied to initialize the pose map using the 6D motion transformation relationship obtained from the front end. Then, closed-loop detection is carried out to add closed-loop constraint conditions, and the non-linear error function optimization method is used for pose optimization. Finally, the global optimal pose, camera trajectory, and reconstructed 3D map environment are obtained.

### 3.2. Shortcomings of the Original RGB-D SLAM Algorithm

The original RGB-D SLAM framework is shown in Figure 1. It can also be improved in terms of efficiency, real-time, and accuracy. In the stage of feature detection and feature extraction of the original algorithm, the methods in common use are the SIFT, SURF, and BRISK algorithms. Although the SIFT and SURF algorithms have higher scale invariance and affine invariance compared with the traditional algorithms, there are still some shortcomings that cannot be ignored, such as the distribution of the extracted feature points is not uniform enough, dependence on the main direction in the process of feature extraction is high, the time for computing is longer, and point pairs that matched correctly are less. The BRISK algorithm is not stable in terms of scale invariance and rotation invariance. In the feature matching stage, the brute force algorithm is usually used, but its efficiency is low and the error in the matching process is large, so the rate of a successful match is low. In the process of motion transformation optimization, the ICP algorithm [15] also has shortcomings. It is easy to fall into the local maximum. ICP algorithm relies heavily on the initial registration location, which requires the initial location of two point clouds to be close enough, and may lead to registration failure when there are noise points and external points. When the pose graph is optimized, the g${}^{2}$o is a C++ project managed by cmake that its description of the document is less and more complex.

Because of these shortcomings, the efficiency of the whole RGB-D SLAM algorithm is reduced, and the real-time performance and accuracy need to be strengthened. Therefore, the original RGB-D SLAM algorithm is improved in Section 4.

## 4. Improvement on the Original RGB-D SLAM Algorithm

We improve the RGB-D SLAM algorithm from three aspects: feature extraction, 3D point cloud registration, and pose optimization. Feature extraction has been introduced in paper [12]; this section will introduce 3D point cloud registration and pose optimization.

### 4.1. 3D Point Cloud Registration Based on SVD Algorithm

The registration problem of point clouds [50] in multi-view is to put the point clouds obtained by Kinect from different viewpoints into the same spatial coordinate system by rotation and translation. So, the core of the problem is to solve the transformation matrix between every two points cloud. In this paper, the BRISK_D algorithm [12] is used to extract feature matching pairs of 2D RGB images, and the RANSAC algorithm is used to filter mismatching pairs. The remaining matching pairs are mapped to the spatial coordinate system and the SVD (singular value decomposition) algorithm [51] is used to solve the transformation matrix of point cloud registration.

After extracting the local features, the remaining important work is how to solve the scanning pose relationship of two adjacent frames or two scenes, that is, the local coordinate corresponding information scanned by the sensor at different positions or different positions in the same position. To explain this problem intuitively and quickly, we assume that the Kinect sensor scans a scene A at the position *a* and a scene B at the position *b*, the two-dimensional image and three-dimensional point cloud information represented by scene A and scene B belong to the local coordinate system with position *a* and position *b* as the coordinate origin, respectively. The key of the problem is how to find the coordinate correspondence between the two coordinate systems, and adjust the data of the two scenes to the same coordinate system. Because the two scenes are in two different coordinate systems, the focus of our research is to calculate the transformation matrix between scenes. The so-called transformation matrix is actually the transformation relationship of scene data from one coordinate system to another, namely rotation and translation. For the sensor kinect, the depth image data can correspond to the color image data after the depth image registration. Therefore, this provides us with an idea: the problem that is difficult to solve at the 3D point cloud level can be first transferred to the 2D image level, and then returned to the 3D point cloud after the processing ends. Therefore, in solving the transformation matrix, after extracting the local features of two adjacent images, the feature matching pair can be mapped to the three-dimensional point cloud coordinate pair, and then the position transformation relationship between the two scenes can be calculated, that is, the rotation (R) and the translation (T) matrix between the two coordinate systems.

In this section, we use the SVD algorithm to calculate the sum matrix between two scenes. This algorithm was first proposed by Arun et al. in 1987 [52] and used in the least square fitting of 3D point cloud set data. The selected 2D point set of the filter is mapped to the H-dimensional space by RANSAC matching [53] to form two point cloud sets $P=\{p_{i}\}$, $Q=\{q_{i}\}$ (i=1,2,3,⋯N; $p_{i}$, $q_{i}$ is 3 × 1 matrix). The relationship between *P* and *Q* can be expressed by Equation (1)

(1) Q=RP+T+N

where R is a 3 × 3 rotation matrix, T is a translation matrix, and N represents the amount of noise interference. R and T are solved by the least-squares method of Equation (2)

(2) $$f\left(R,T\right)=\sum_{i=1}^{n}\left|\right|q_{i}-Rp_{i}-T|{|}^{2}=\min$$

where the centroid of $\left\{q_{i}\right\}$ and $\left\{p_{i}^{\prime }=Rp_{i}+T\right\}$ is the same. The centroid of $\left\{q_{i}\right\}$ and $\left\{p_{i}^{\prime }=Rp_{i}+T\right\}$ is solved by the following equation

(3) $$q=\frac{1}{N}\sum_{i=1}^{N}q_{i},$$

(4) $$p=\frac{1}{N}\sum_{i=1}^{N}p_{i},$$

(5) $$p^{\prime }=\frac{1}{N}\sum_{i=1}^{N}p_{i}^{\prime }=Rp+T,$$

where $q=p^{\prime }$

By translating the two-point cloud sets $P=\{p_{i}\}$, $Q=\{q_{i}\}$, two new 3D point cloud sets are represented as follows

(6) $$m_{i}=p_{i}-p,$$

(7) $$m_{i}^{\prime }=q_{i}-q$$

After corresponding changes, we get the expression $\sum_{i=1}^{N}{\left({m^{\prime }}_{i}-Rm_{i}\right)}^{2}$. The rotation matrix *R* is obtained by solving the minimum of the expression.

Equation (2) is derived as follows

(8) q=Rp+T

where the translation matrix T=q−Rp.

The 3D point cloud registration based on the SVD algorithm is shown in Algorithm 1.

**Algorithm 1** 3D point cloud registration based on SVD algorithm**Input:** Two-point cloud sets: $P=\{p_{i}\}$, $Q=\{q_{i}\}$, i=1,2,3,⋯,N **Output:**
$f\left(R,T\right)=\min\sum_{i=1}^{n}{\left(q_{i}-Rp_{i}-T\right)}^{2}$1:Calculate the centroid of two 3D point cloud sets $P=\{p_{i}\}$, $Q=\{q_{i}\}$ according to Equations (4) and (5);2:Translating the two point cloud sets $P=\{p_{i}\}$, $Q=\{q_{i}\}$ to get the two new 3D point cloud sets $\left\{m_{i}\right\}\left\{m_{i}^{\prime }\right\}$ according to Equations (6) and (7);3:Computing 3 × 3 matrix $C=\sum_{i=1}^{N}m_{i}{{m^{\prime }}_{i}}^{T}$;4:Decomposition of singular values for matrix *C*, $C=U\Lambda V^{T}$, where $\Lambda =diag\left(d_{i}\right),d_{1}\geq d_{2}\geq d_{3}\geq 0$ and the rotation matrix $R=VU^{T}$;5:Computing the translation matrix T=q−Rp.


However, there is still a very important problem in the solution process, that is, how to select four feature matches. It is not difficult to find through many experiments that there are many feature matching pairs where the position is concentrated. Once one of these matching pairs is selected to solve the rotation matrix R and the translation matrix T, it is likely that the matrix R and T only satisfy these parts. The concentrated feature points are gathered, and the feature point clouds which are far away from each other cause a large error, that is to say, there is a phenomenon that the local matching is good but the global fusion is poor.

Therefore, when selecting four feature matching pairs, we should choose the global dispersion as much as possible. In this paper, we use Euclidean distance to select matching pairs. The maximum Euclidean distance between the matching pairs on the same 2D image is calculated. If the distance between any two of the four matching pairs on the same 2D image is greater than 0.5 times the maximum Euclidean distance, the four feature matching pairs are selected to transform into 3D space. By calculating the rotation and translation matrices through the above five steps, the registration of three-dimensional point clouds can be realized.

### 4.2. Optimization of Pose Graph Based on HOG-Man

We can suppose $x={\left(x_{1},x_{2},\cdots ,x_{n}\right)}^{T}$ is a vector of robot’s poses, where $x_{i}$ is the pose at node *i*, $z_{ij}$ and $\Omega_{ij}$ refer to the average value and information matrix of the jointly observational node *i* and *j*, respectively. ${\hat{z}}_{ij}\left(x_{i},x_{j}\right)$ indicates estimated value at $x_{i}$ and $x_{j}$. So, we can define an error function $e\left(x_{i},x_{j}\right)$ to calculate the difference between ${\hat{z}}_{ij}$ and $z_{ij}$: $e_{ij}\left(x_{i},x_{j}\right)=z_{ij}-{\hat{z}}_{ij}\left(x_{i},x_{j}\right)$. The purpose is obtaining the node $x^{*}$ and making the negative log-likelihood $F\left(x\right)$ of the set of observation data to come to the minimum. In machine learning, it is accustomed to using the optimization algorithm to find the minimum value, so the negative log-likelihood (NLL) is usually used. As the probability is always less than or close to 1, the value of NLL is always greater than 0. The probability is close to 1, and the value of NLL is close to 0, reaching the minimum value.

The maximum likelihood method is used to obtain the pose of node $x^{*}$ and minimize the negative log-likelihood function $F\left(x\right)$ of all observations. $F\left(x\right)$ is expressed by Equation (9).

(9) $$F\left(x\right)=\sum_{\langle i,j\in C\rangle }\underset{F_{ij}}{\underbrace{e_{ij}^{T}\Omega_{ij}e_{ij}}}$$

where *C* is a set of exponential pairs that restricts the existence of *z*. Therefore, the objective of optimizing the pose graph is

(10) $$x^{*}=\arg\min_{x}F\left(x\right).$$

The HOG-Man algorithm is an effective method to solve the target.

#### 4.2.1. Hierarchical Pose-Graph

**Figure 2:**
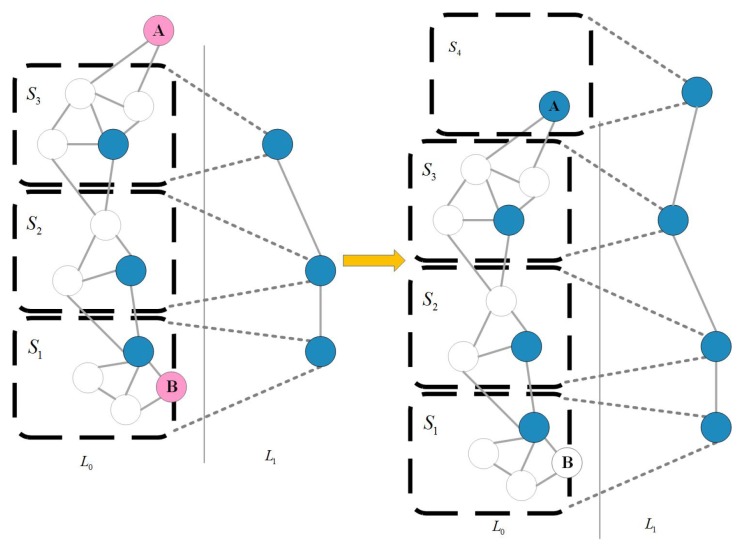
Updating process of the hierarchical pose-graph.

The construction process of a hierarchical pose-graph is an incremental process. The core of this idea is, according to some standards, thus: the original graph is divided into multiple sub-graphs and one node of a sub-graph is used to represent the sub-graph to get the abstraction of the original layer. Then the multilevel graph structure is obtained by abstracting the resulting graphs in proper sequence, which can extract the topology structure of the original graph. When a new node is added to the diagram, it is first added to the original graph, and then it can be judged whether the added node changes the sub-map division. If there is a change, it needs to update the high-level map and simultaneously optimizes the top-level, the underlying diagram will be updated and the reverse transmission will go on from the top of the map to the bottom layer only when the topological structure of the top-level makes a great change. This ensures real-time optimization. The core of the process is the representation problem of abstraction at different levels. Each layer is a pose map and there is a corresponding relationship between the abstraction layers. The updating process of the hierarchical pose-graph is shown in Figure 2.

This establishment process is an incremental process. Figure 2 illustrates the updating process of the hierarchical pose-graph by taking a two-layer graph as an example. Suppose you have the graph structure on the left in Figure 2. Among them, *S* represents the subgraph, *L* represents the abstract level, the white points represent the $L_{0}$ layer nodes, and the blue points in the $L_{0}$ layer are selected as the $L_{1}$ layer nodes. There are already three subgraphs on the left. When two new nodes like red node A and node B need to be added, the distance between the node and the representative node in the nearest subgraph is used to determine whether to add an existing subgraph (such as node B) or establish a new subgraph (such as node A) on the right in Figure 2.

#### 4.2.2. Linearized State Space as a Manifold

We can assume the initial estimation of the robot’s position as $\overset{˘}{x}$. Equation (10) can be solved by Gauss-Newton or Levenberg-Marquardt. This idea is to approximate the error function through the first-order Taylor expansion of the initial estimation $\overset{˘}{x}$

(11) $$\begin{array}{ccc} e_{ij}\left({\overset{˘}{x}}_{i}+\Delta x_{i},{\overset{˘}{x}}_{j}+\Delta x_{j}\right) & = & e_{ij}\left(\overset{˘}{x}+\Delta x\right)\\ & ≃ & e_{ij}+J_{ij}\Delta x \end{array}$$

where $J_{ij}$ is a Jacobian of $e_{ij}$, which can be solved by $\overset{˘}{x}$, $e_{ij}\overset{\mathrm{def}}{=}e_{ij}\left(\overset{˘}{x}\right)$. Substituting Equation (11) into Equation (9):

(12) $$\begin{array}{ccc} F_{ij}\left(\overset{˘}{x}+\Delta x\right) & = & e_{ij}{\left(\overset{˘}{x}+\Delta x\right)}^{T}\Omega_{ij}e_{ij}\left(\overset{˘}{x}+\Delta x\right)\\ & = & {\left(e_{ij}+J_{ij}\Delta x\right)}^{T}\Omega_{ij}\left(e_{ij}+J_{ij}\Delta x\right)\\ & = & \underset{c_{ij}}{\underbrace{e_{{}_{ij}}^{T}\Omega_{ij}e_{ij}}}+\underset{b_{ij}}{\underbrace{2e_{{}_{ij}}^{T}\Omega_{ij}J_{ij}}}\Delta x\\ & & +\Delta x^{T}\underset{H_{ij}}{\underbrace{J_{{}_{ij}}^{T}\Omega_{ij}J_{ij}}}\Delta x\\ & = & c_{ij}+2b_{ij}\Delta x+\Delta x^{T}H_{ij}\Delta x. \end{array}$$

According to the principle of local approximation, F(x) can be represented as

(13) $$\begin{array}{ccc} F\left(\overset{˘}{x}+\Delta x\right) & = & \sum_{\langle i,j\rangle \in C}F_{ij}\left(\overset{˘}{x}+\Delta x\right)\\ & ≃ & \sum_{\langle i,j\rangle \in C}c_{ij}+2b_{ij}\Delta x\\ & & +\Delta x^{T}H_{ij}\Delta x\\ & = & c+2b^{T}\Delta x+\Delta x^{T}H\Delta x \end{array}$$

where in Equation (13), $c=\sum c_{ij}$, $b=\sum b_{ij}$ and $H=\sum H_{ij}$. We can minimize $\Delta \tilde{x}$ through the linear system solution

(14) $$H\Delta x^{*}=-b$$

where *H* is the information matrix of the system. Add $\Delta x^{*}$ to the robot’s initial pose estimation $\overset{˘}{x}$:

(15) $$x^{*}=\overset{˘}{x}+\Delta x^{*}.$$

Equation (13) is iterated using a Gaussian-Newton method to solve Equation (14) and update Equation (15). In the iterative process, the solutions which have been obtained previously are served as linearized reference points and initial estimation [53].

Manifold is a space with local Euclidean space property. It is used to describe the geometric form in mathematics [54]. In the SLAM, each parameter $x_{i}$ is made up of a translation component $t_{i}$ and a rotation component $\alpha_{i}$. Among them, translation components exist in Euclidean space, but rotation components span 2D and 3D spaces. First, considering that the state space is not a Euclidean space, but the manifold allows us to handle singular points by introducing angular components. With manifolds, we can find a more efficient linearization system to solve the optimization problem.

We can assume Θ as an operator, which represents the mapping of local variable Δx from Euclidean space to a manifold xΘΔx. Then, based on this process, the error can be expressed by the following new equation

(16) $$\begin{array}{ccc} e_{ij}^{\overset{˘}{x}}\left(\Delta {\tilde{x}}_{i},\Delta {\tilde{x}}_{j}\right) & \overset{\mathrm{def}}{=} & e_{ij}\left({\overset{˘}{x}}_{i}\Theta \Delta {\tilde{x}}_{i},{\overset{˘}{x}}_{j}\Theta \Delta {\tilde{x}}_{j}\right)\\ & = & e_{ij}\left(\overset{˘}{x}\Theta \Delta \tilde{x}\right)\\ & ≃ & e_{ij}+{\tilde{J}}_{ij}\Delta \tilde{x} \end{array}$$

where

(17) $${\tilde{J}}_{ij}=\frac{\partial e_{ij}\left(\overset{˘}{x}\Theta \Delta \tilde{x}\right)}{\partial \Delta \tilde{x}}{|}_{\Delta \tilde{x}=0}.$$

By substituting Equation (16) into Equations (13) and (17), the following equation is obtained

(18) $$\tilde{H}\Delta {\tilde{x}}^{*}=-\tilde{b}.1$$

$\Delta x^{*}$ is an increment, which can be calculated. Combining with the operator Θ, Equation (15) can be updated.

(19) $$x^{*}=\overset{˘}{x}\Theta \Delta {\tilde{x}}^{*}.$$

To summarize, the problem of optimization has been solved by combining Equations (18) and (19).

## 5. Design of the RGB-D SLAM Algorithm Combined with Cloud Robot

The implementation of the RGB-D SLAM algorithm in the cloud robot framework is to overcome the high complexity, such as the huge amount of data, complex computation, and so on. However, due to the limitations of the SLAM algorithm and the network delay, it is not feasible to move all calculations to the cloud. To solve this problem, the robot client sends the key frame to the cloud server through a standard wireless network and completes the tracking task with the local sub-optimal Map. While the cloud server constructs the global map after receiving the key frame, and also optimizes and fuses the local map to the global map, then sends the final map to the robot client.

### 5.1. Framework of Cloud Robot

The visual RGB-D SLAM method proposed in this paper is mainly based on a distributed framework, which takes expensive computing and storage tasks as a service in the cloud, while those tracking tasks with high real-time requirements can be used as local client services. In this way, mobile robots can be freed from complex computational tasks in the process of motion, and more computational resources can be allocated to tasks with higher real-time requirements, such as obstacle avoidance, navigation, and tracking.

**Figure 3:**
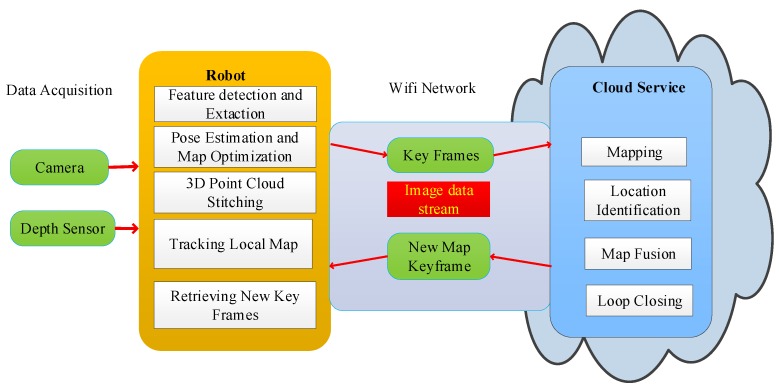
Framework of Combining RGB-D SLAM with Cloud Robot.

In terms of cloud robotics, researchers have proposed many algorithms for the SLAM problem. The three most popular frameworks are DAvinCi, Rapyuta, and C${}^{2}$TAM. This paper combines the RGB-D SLAM algorithm with the framework of C${}^{2}$TAM. The improved framework is shown in Figure 3.

In the data acquisition part, a variety of sensors can be chosen, such as a camera, depth sensor, stereo vision sensor, and so on. 3D spatial information, such as RGB image information and depth information, is acquired by visual sensors. After the information is collected by the robot, this part of the work that information is processed by the robot is simply referred to as the tracking work. The tracking work includes the task of feature detection and descriptor extraction, pose estimation and pose map optimization, 3D point cloud stitching, tracking local map, and retrieving new key frames.

In the tracking work, the camera pose estimation is completed, the camera pose state $\tau^{t}$ at each time *t* is estimated, and the local map tracking is completed. At the same time, the key frames are sent to the cloud. Different from the previous SLAM method, the mapping part is put into the cloud and the standard wireless network is used to connect in the middle process. At the same time, the local robots and the cloud servers share the map information.

In the improved algorithm, the robot client sends a new key frame to the cloud map construction part only when the collected information contains new map information during the tracking process. According to the received new key frames the map construction thread in the cloud matches the feature points corresponding to the same scene between different key frames. After matching, the 3D scene positions of these scenes are restored by triangulation. BA adjustments are made to all key frames and 3D points, and then the accurate three-dimensional map is restored.

After each BA adjustment, the new map is sent to the robot. The frequency of information transmission between the robot client and the cloud server is lower than the normal frequency of data acquisition. In the exploratory stage, the number of new key frames is very small, far less than the frame rate, and there will be no new key frames generated in the places that have already passed. During the experiment, the standard wireless network can be used to connect. Tracking and map building tasks can be completed on different computers. In addition, it has high robustness for network delay in the two data streams. The tracking part can run sub-optimal maps until the next globally optimized map is completed and sent, which can reduce the loss of camera tracking and ensure the normal tracking process.

In the improved SLAM algorithm, map optimization and storage functions with a high computational cost and low real-time requirements are allocated to the cloud. Tracking tasks with high real-time requirements are allocated to run on local robots, and only a reliable communication network is needed in the middle. Because of the low traffic between the cloud and robot client, a standard wireless communication network can meet the communication requirements in this paper.

### 5.2. RGB-D SLAM Algorithms with Cloud Robot

#### 5.2.1. Separation of Tracking and Map Construction

During the tracking process, key frames and feature points in the image are extracted by modeling each frame image and described by descriptors, and then the position state of the camera is obtained through the feature point change relationship between different frames

$$\tau^{t}=\left\{T,d_{t}\right\}$$

Through the transformation of the position state, the sequence of the pose transformation relationship of the camera is obtained, and then the pose is estimated to realize the tracking process. In this process, it is often carried out at a certain frame rate. If some frames are skipped, tracking failure may occur. Therefore, the real-time requirement of the tracking process is relatively high. It is sensitive to network delay, and the amount of data is relatively large, so it is not suitable for processing in the cloud. However, it can be used as a local service, and the distribution is run on the local machine. Through image tracking, the robot can extract the key frames and transmit them to the cloud server.

In the map construction, scene maps are estimated according to the key frame scenario model extracted by the tracking part. At the same time, different maps in the map database $\left\{M_{1},\ldots ,M_{k},\ldots ,M_{l}\right\}$ are optimized. The scene features shared in some key frames are merged to form the global optimal map. The tasks, such as estimation, optimization, and map fusion in map construction are very computationally intensive and usually, take up more than half of the SLAM problem. Although it consumes a lot of computing resources, there are no very high real-time requirements for optimization problems in the database.

In the process of tracking, it is possible to lose the tracking because a frame of the image is not processed. However, the optimization process is for the whole and does not affect the backend work because one frame of image is not processed. During the optimization process, the key frame BA method can be selected to optimize the map, which can reduce the communication between the robot client and the cloud.

At the same time, the tracking part can also use sub-optimal maps to track, so that the results of tracking will not be affected by ongoing map construction. With the above features, the map construction part can tolerate network delay, so it can be used as a cloud service. At the same time, through the huge data processing and storage capacity of the cloud server, it can effectively save data processing time, reduce the load of the robot, and reduce the weight of the robot.

Robot client and cloud servers are connected through the standard Wifi network. The robot client sends key frames to the cloud. The map building part of the cloud receives key frames, and optimizes the key frames to complete the map building. Because key frames need to be detected and extracted by the feature, the image frame can be extracted as a key frame only if new feature information is included in the newly received image frame. Therefore, the ratio of key frames is very low compared to the total image frame. In other words, in the process of transmission, only the key frames need to be transmitted to the cloud, rather than all the image frames needing to be transmitted to the cloud. This can greatly reduce the bandwidth load, ensure the communication speed between the robot and the cloud, ensure the real-time performance of the robot, and achieve the separation of tracking and map building.

#### 5.2.2. Location Recognition and Relocation Separation

The mobile robot relocates the place where it has been estimated and saved in the cloud. This process is called relocation. The relocation in the tracking process is mainly reflected in two aspects:

(1) The system worked smoothly during the tracking process but the system tracking is lost due to sudden movement or large occlusion. In this case, the system is likely to be on the original map. Therefore, the system relocation process only needs to search the current map and this part of the process only needs to be executed on the local robot.

(2) Robots have been lost for a long time or have just started. In this case, the robot relocation needs to search for more relevant maps.

For (1), the relocation can be run on the robot itself because the robot has its own sub-optimal local map. For (2), map search in a large map environment is very computationally intensive, so it can be allocated as a cloud service in the cloud.

However, this process is particularly sensitive to network latency. If the self-localization process takes too much time, the robot may have moved to another place. Therefore, the self-positioning process needs to solve the problems of large data search and sensitivity to network delay. During the experiment, a small image comparison may cost about 0.03 ms, but there are hundreds of image frames in a map and hundreds of maps in a database. Therefore, a search can easily take several seconds. In a few seconds, the robot can move for a long distance.

To solve the above problems, this paper proposes to separate the location recognition and the self-localization, the location recognition can run in the cloud the self-localization can run in the robot client.

**Figure 4:**
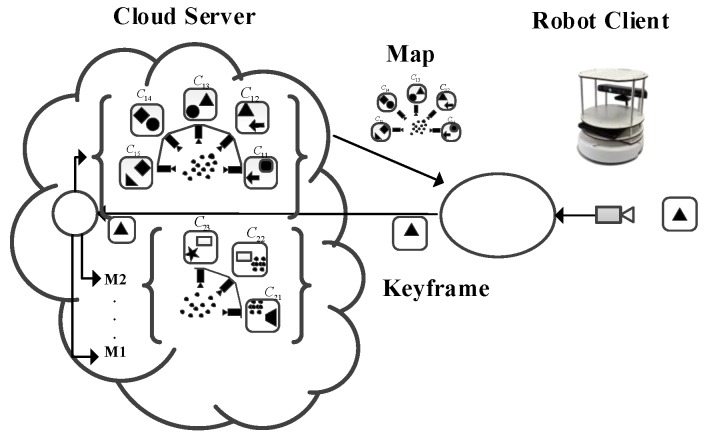
Data transfer problem in position recognition.

**Figure 5:**
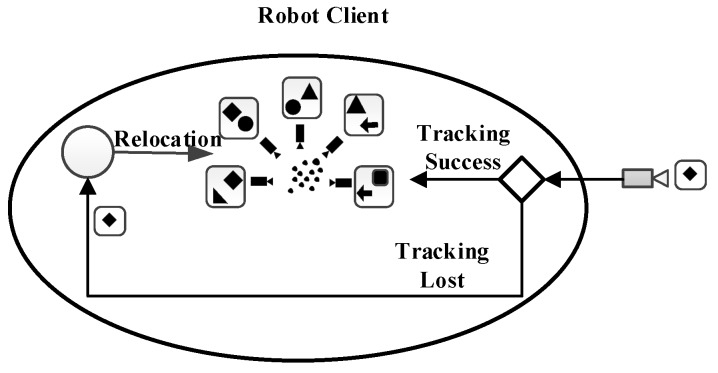
Client’s relocation process.

(1) The Location Recognition: the cloud server quickly and roughly searches the map. This fast search takes a lot of time, and the camera may have moved a distance when the relocation data arrives at the client. Therefore, after the retrieval is completed, the cloud server sends a small number of filtered relocation candidates to the client, including the key frames closest to the first stage and several key frames from the same map. The process is shown in Figure 4. The robot client sends key frames to the cloud, and the cloud retrieves the key frames. After finding the corresponding map, the key frames corresponding to the map are transmitted to the robot client, which contains some key frames adjacent to the current key frame. Even if the robot has moved, it can be relocated to the position of the robot through the surrounding key frames.

(2) Relocation: When the client receives the key frames sent by the cloud server, it finds the corresponding sub-optimal local map, and relocates the key frames acquired by the current robot, and locates them accurately. Therefore, the delay does not affect the second stage, as shown in Figure 5.

#### 5.2.3. Cloud Map Fusion

**Figure 6:**
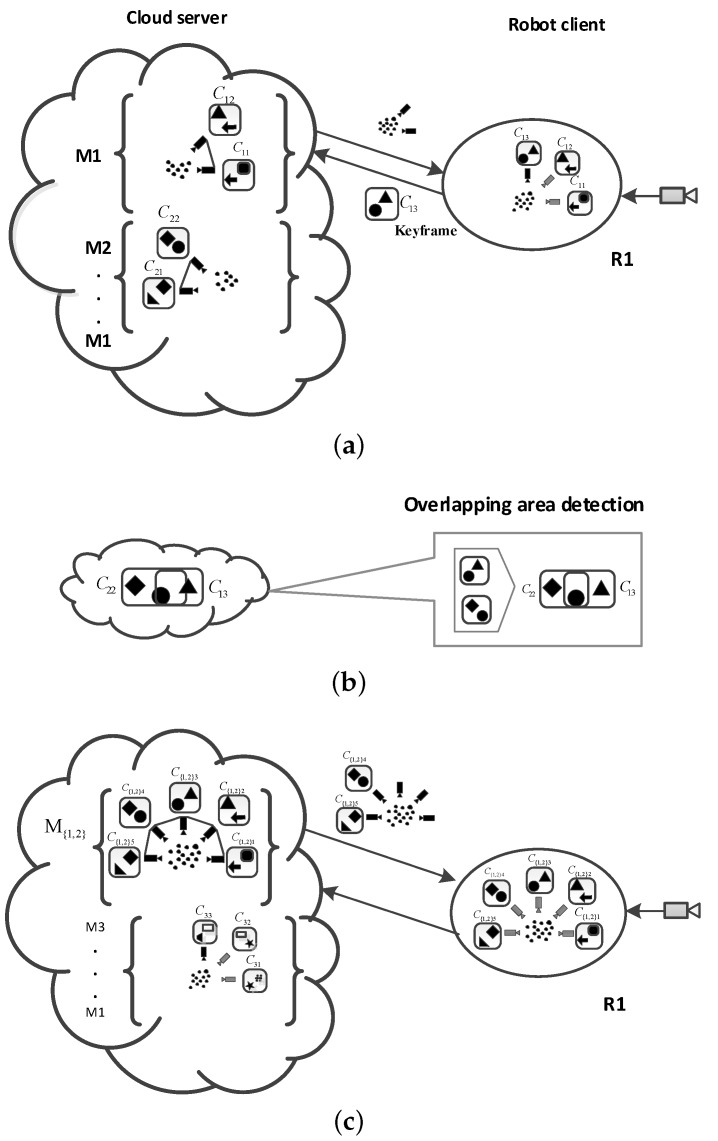
Map fusion process in cloud services. (**a**) Update keyframe to the cloud; (**b**) Overlapping detection; (**c**) Send New map to the robot client.

After receiving the key frames, the cloud server retrieves all the key frames. If common features are found, there are the overlapping areas of the map. In fact, the process of cloud map fusion is to splice and merge the common features of different maps, to splice the common features of the maps, and to construct a global map with more consistency through splicing and fusion. Because there is no real-time limitation in the process of map fusion, the process of map optimization and fusion in SLAM can be performed in the cloud. The complete map fusion process can be seen in Figure 6. Cloud map fusion can not only improve the efficiency of key frame search and fusion but also integrate different maps constructed by different robots into globally consistent maps, thus realizing multi-robot map construction.

In Figure 6a, The robot client $R_{1}$ tracks the camera pose and uploads a new keyframe $C_{13}$ to the cloud, which builds map $M_{1}$ of newly uploaded key frames. At the same time, the cloud compares the newly uploaded key frames and detects the overlapping parts of the map, such as $C_{13}$ in $M_{1}$, and $C_{22}$ in $M_{2}$ in Figure 6b. After finding the overlapping part, the overlapping part is merged to form a new map $M_{12}$. At the same time, the cloud sends the new map $M_{12}$ to the robot client, as shown in Figure 6c. In this process, high communication traffic may be generated because the robot does not have optimized maps and keyframes, so the map $M_{12}$ and keyframe feature points need to be sent to the robot client. Although the process may generate high traffic, the real-time requirement of the process is not high, so it can be sent slowly. In this way, local maps are updated to new maps.

At the same time, it takes a lot of time to download the map $M_{12}$. In order to ensure the robustness of the algorithm, the following measures can be taken:

(1) When the map $M_{k}$ is sent from the server to the client, the key frames should also be downloaded to the client. The transmission order of key frames can be determined by the key frames and the current frame of the camera. The key frames with smaller distances are sent first. This ensures that the client has the best possible estimate of the currently accessing area, while the remote area does not interfere with the current estimate.

(2) When the map $M_{k}$ is downloaded, the client can explore new areas and upload new key frames to the cloud server. But these new keyframes may take a long time to be downloaded because the map $M_{k}$ has to be downloaded first. The solution to this problem is that the server performs a local BA adjustment at any time. When the key frames arrive and send them to the high priority clients. Through this method, we can ensure that the initial key frame can be sent to the client quickly and that the tracking is not lost again.

## 6. Experiments

We divide the experiments into three parts. The first part compares the original RGB-D SLAM algorithm with the improved RGB-D SLAM algorithm. The second part is the experimental analysis of the RGB-D SLAM algorithm combined with cloud robotics. The third part compares the two different methods of local RGB-D SLAM and RGB-D SLAM combined with the cloud robot in multiple indoor scenes.

Similar to the C${}^{2}$TAM framework, we use ROS(Robot Operating System) to realize our cloud computing platform for RGB-D SLAM. The robot client is a laptop (Intel Core i7-4700HQ CPU and 8.0GB RAM) which runs the tracking process. The cloud server is a Linux virtual machine(Ubuntu Server 16.04 LTS 64bit) running on our university’s private cloud which enables ubiquitous on-demand access to a shared pool of configurable computing resources. They are connected through our university’s wireless LAN. The private cloud is deployed on a high-performance server cluster which including a management node and 7 computing nodes. The ROS middleware is used throughout our system (the cloud server and the robot client) to provide tools for managing global access to parameters and data.

In order to test the validity of our algorithm, we implement two kinds of experiments. The first kind is conducted online in our lab environment, and a Microsoft Kinect (V1) is connected to the robot client providing 640 × 480 RGB and depth frames at a 30-Hz rate. The second kind is accomplished off-line by utilizing the RGB-D benchmark provided by the Technical University of Munich [55]. The Benchmark data were taken also by a Microsoft Kinect (V1) sensor, and the ground truth data were taken by a highly accurate motion capture system which was composed of eight 100-Hz cameras.

### 6.1. Comparison of the RGB-D SLAM Algorithm

**Figure 7:**
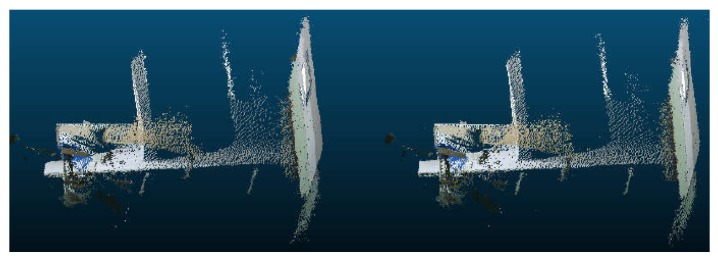
The point clouds to be matched.

**Figure 8:**
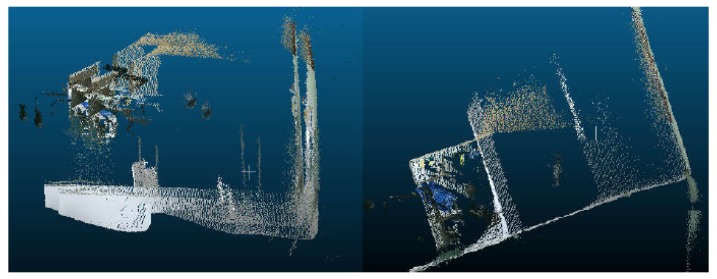
The registration effect of two different methods.

Before introducing the effect of the RGB-D algorithm, we first compare ICP and SVD registration methods through four sets of point cloud scenarios. These experiments are completed on the robot. Figure 7 and Figure 8 show the experimental results of one set of data. Figure 7 is the point cloud data to be matched. The left side of Figure 8 is the registration result of point clouds using the ICP algorithm, and the right is the registration result obtained by the SVD method. We find that the ICP matching results are biased, the effect is general, and the SVD matching edge features tend to be consistent, the effect has been significantly improved. It shows the effectiveness and practicability of the SVD algorithm.

From the data in Table 3, we can see that the traditional ICP algorithm has 50 iterations and the registration time is 60,118 ms. The improved SVD algorithm has 10 iterations and the registration time is 7183 ms. From the point of view of registration error, the registration error of this algorithm is smaller than that of the traditional ICP algorithm. Therefore, it can be concluded that the proposed algorithm not only shortens the registration time but also has a smaller registration error and higher registration accuracy.

**Figure 9:**
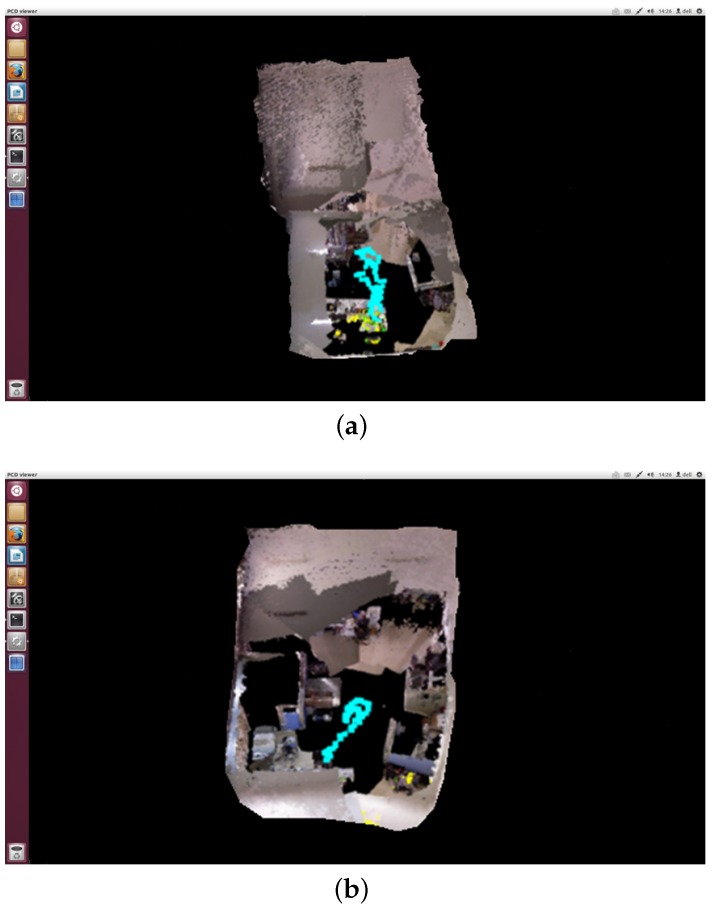
Comparison of two RGB-D algorithms. (**a**) A 3D map obtained by using the original RGB-D SLAM algorithm. (**b**) A 3D map obtained by using the improved RGB-D SLAM algorithm.

Figure 9a,b show our lab environment, which can better verify the effectiveness and timeliness of our algorithm. Figure 9a is a 3D map obtained by using the original RGB-D SLAM algorithm. It can be seen from the map that there is a large error in the horizontal and vertical directions, and in the process of stitching, there are double shadows and bending, resulting in low accuracy of map building. Figure 9b is a 3D map obtained by using the improved RGB-D SLAM algorithm. As can be seen from the map, the stitching effect of the map has been greatly improved, and the problem of the double shadows and bending is also solved, which also shows that the cumulative error in the map building process is reduced. Thus, the need for high precision and high quality is achieved. Through the analysis of two figures, we can see that the improved algorithm can better complete the stitching of the map indoor and then combine the evaluation effect of each link, it fully proves that the improved algorithm is more advantageous.

**Figure 10:**
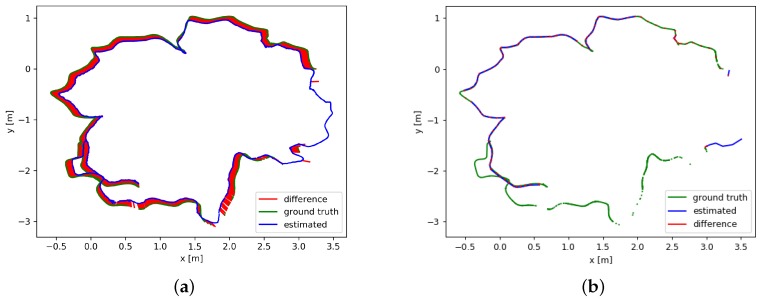
Comparison of the trajectories for the sequence “freiburg2_room”. (**a**) The trajectories obtained by using the original RGB-D SLAM algorithm. (**b**) The trajectories obtained by using the improved RGB-D SLAM algorithm.

Figure 10 shows the trajectory results of the sequence “freiburg2_desk” compared to the ground-truth with two algorithms. Figure 10a shows the results of the original RGB-D SLAM. The green trajectory represents the ground truth. The difference between the estimated trajectory and the ground truth trajectory is shown in red. Figure 10b shows the results of the improved RGB-D SLAM algorithm. To evaluate the different algorithms, we use the absolute trajectory error (ATE) as proposed by Sturm et al. [21]. Table 4 shows the statistical results of trajectory error indicators. The six trajectory error indicators are RMSE(Root mean squared error), Mean, Median, STD(Standard Deviation), MIN and MAX. The results show that the location error of our improved algorithm is obviously reduced.

### 6.2. Experimental Analysis of the RGB-D SLAM Algorithm Combined with Cloud Computing

In this section, we analyze the computing time consumed by the cloud server and the robot client, and the network flow between the cloud server and the client. At the same time, we analyze the cloud map merging. The experiment deals with the “freiburg2_desk” sequence in the TUM RGB-D Dataset, an RGB-D sequence of 2964 frames which was recorded at full frame rate(30 Hz), and the sensor resolution is 640 × 480, using the proposed algorithms.

#### 6.2.1. Computational Performance and Bandwidth Analysis

**Figure 11:**
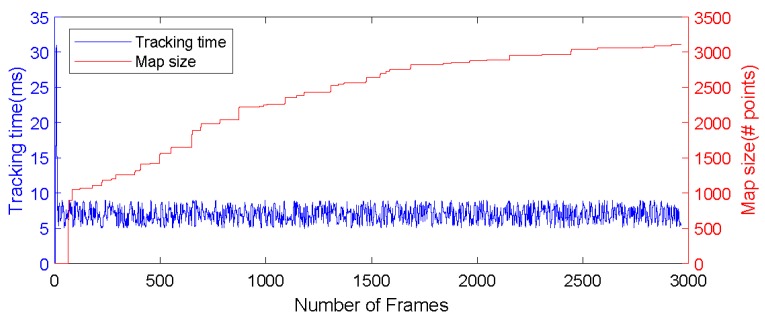
Computational performance analysis (map size (right axis) and tracking time (left axis)).

During the experiment, all experiments can be run in real-time, and the devices in the experiment are connected to our university’s wireless LAN. Figure 11 shows in a double-axis figure the computational performance of the robot with the tracking process and the size of the constructed map. It can be seen from Figure 11, the horizontal axis represents the number of tracking image frames, and the vertical axis represents the tracking time (in blue) and the size of the generated map (in red). The computation time of the tracking process is kept at about 8 ms. Even if the tracking is lost, it can be kept within a certain threshold.

This shows that the tracking part can be completed on the robot’s onboard computer with limited local resources while ensuring the real-time performance of the tracking. It should be noted that the calculation in the front part of the sequence is about 30 ms, which is caused by the initialization of the first 25 frames, which does not affect the subsequent results of the experiment. During the entire tracking process, the time consumed by tracking has not changed much, but the size of the map is increasing. More and more information is included in the map. From the red curve in the map, it can be seen that the size of the map is increasing as time goes on.

**Figure 12:**
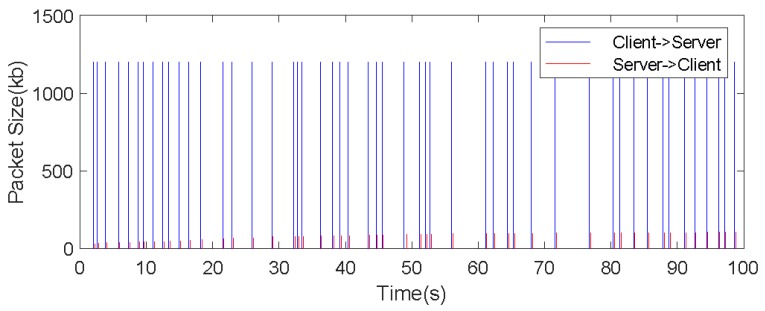
Robot client and cloud server communication analysis.

Figure 12 shows the bandwidth analysis under the same experiment. The blue peak data in the figure indicates that the map builder transmits the data to the cloud server. It can be seen that the heights of the blue peaks are the same because the keyframes sent by the client to the cloud server are the same size.

The red peak indicates the size of data flow information from the cloud server to the client. As can be seen from Figure 12, the size of data flow from cloud to robot increases slightly with the passage of time. This is because in this process, as the range of motion of the robot continues to expand, the map constructed will also continue to expand, and may become a very large related map. At the same time, it also shows that the keyframes and points closer to the current camera pose should be given higher priority, which can keep the client from losing track. Using the proposed algorithm, the map can be constructed in a standard wireless network environment.

Each RGB-D pixel in the keyframe is represented by 4 bytes, and the size of one keyframe is 1200 KB (4B × 640 × 480 = 1200 KB). In the actual communication process, there are pose information, timestamp, and some other parameter information that needs to be transmitted, but these auxiliary messages can be transmitted at no more than 1 kb/s. As can be seen from Figure 12, the average transmission interval of keyframes is more than 1 s, while the average data flow of this experiment is about 1 MB/s, so our algorithm can achieve good real-time results.

#### 6.2.2. Fusion of Overlapping Areas

An important step in the improved algorithm is to achieve the splicing and fusion of the map. The cloud server may contain many maps that are previously reserved. As the robot is re-opened, the relationship between the map and the map is closer. As new keyframes are continuously uploaded to the cloud server, the overlapping areas between the map and the map begin to appear slowly. At this time, the maps containing the common features can be merged by retrieving the common features in the key frames.

**Figure 13:**
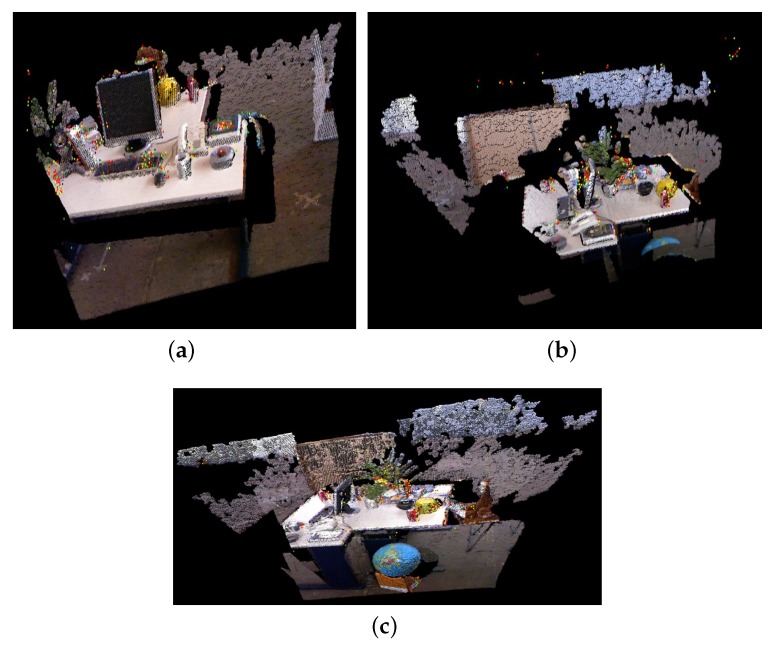
The process of map fusion. (**a**) Map A. (**b**) Map B. (**c**) The map after the fusion.

During the experiment, we assume that in a scene map A, the camera moves from desktop A to desktop B, and the point cloud is composed as shown in Figure 13a. At the same time, there is an estimated map of desktop B in the map of the cloud server, as shown in Figure 13b. When the camera moves from desktop A to desktop B and adds key frames to the map, the similarity between the new key frames and each key frame in other maps is checked to find the common features between the key frames, that is, the common maps with different maps.

Comparing Map A and Map B, it is obvious that there are common areas, such as the computer, the tables, the telephone and the globe in both maps. However, there are no chairs in Map A and no books on the left side of Map A in Map B. For these two maps, it is necessary to fuse common information and preserve their respective non-common features. When the camera moves from A to B, the map fusion process detects the similarity between the current key frame and the previous map key frame, and fuses the two maps into one map, as shown in Figure 13c.

### 6.3. Comparison of Overall Experimental Results

**Figure 14:**
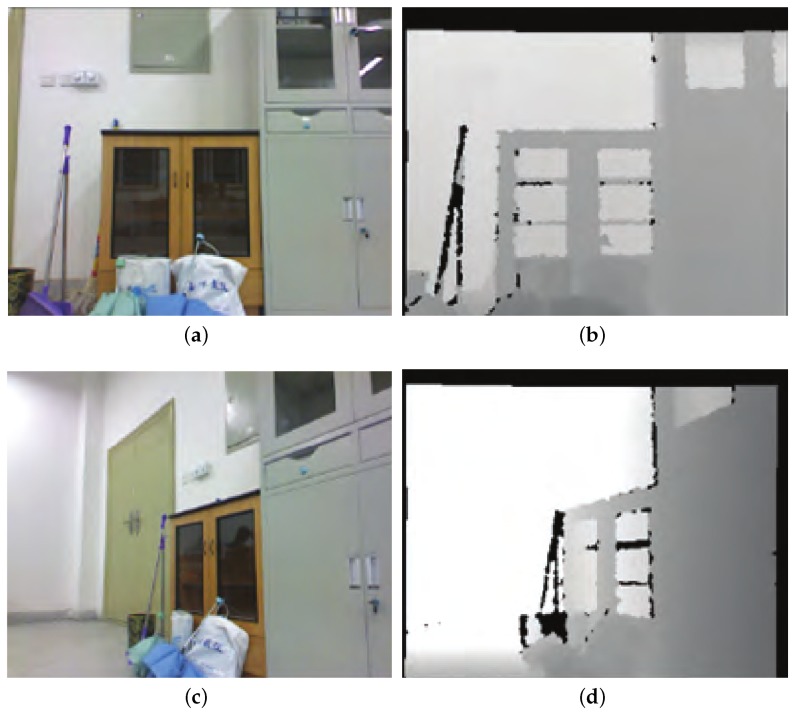
RGB and depth information of two frame images. (**a**) RGB information of the first frame. (**b**) Depth information of the first frame. **(c)** RGB information of the second frame. (**d**) Depth information of the second frame.

In order to verify the effect of the RGB-D SLAM based on cloud robot, we carried out a map building experiment on the 3D environment in the real scene. The RGB information and depth information of the two frame images used in the experiment are shown in Figure 14.

**Figure 15:**
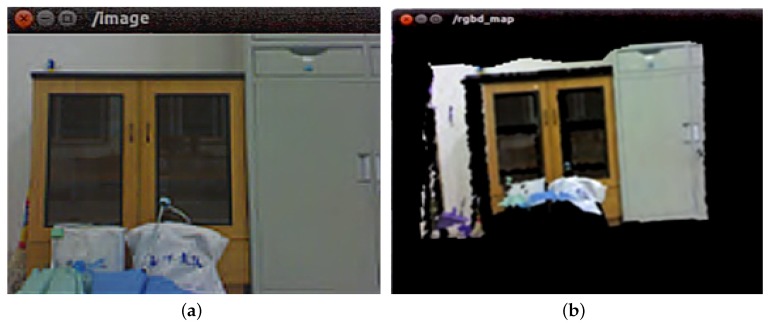
Image display. (**a**) Image displayed for key_frame topic. (**b**) Image displayed for rgbd_map topic.

Figure 15a shows the RGB channel of the key_frame topic which is subscribed by the rgbd_mapping node in the cloud. Figure 15b shows the point cloud channel of the rgbd_map topic which is published by the rgbd_mapping node.

**Figure 16:**
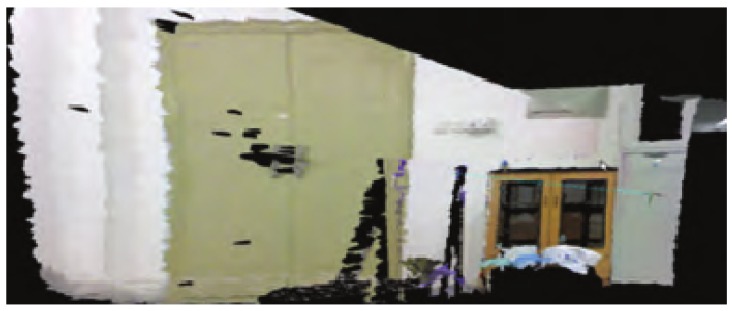
The reconstruction of the 3D environment combined with the cloud.

For the two frames of image data in Figure 14, the reconstruction effect of the 3D environment map using the RGB-D SLAM algorithm combined with the cloud robot is shown in Figure 16. As can be seen from the figure, the reconstruction effect is less deviated from the real environment, and the object in Figure 16 does not produce a ghosting phenomenon, which also proves that the accuracy and stability of the method are relatively high.

We make detailed statistics on the time consumption in the reconstruction of the 3D environmental map in multiple indoor scenes between the two methods, combined in the cloud or not, as shown in Table 5.

It can be seen from Table 5 that the mean time consumption of RGB-D SLAM combined with the cloud is about 12 s, while the local end is around 39 s. As a result, the gap of implementation efficiency is larger for the same scene, which also shows that the method of 3D map building with cloud robot is more effective and more real-time.

**Figure 17:**
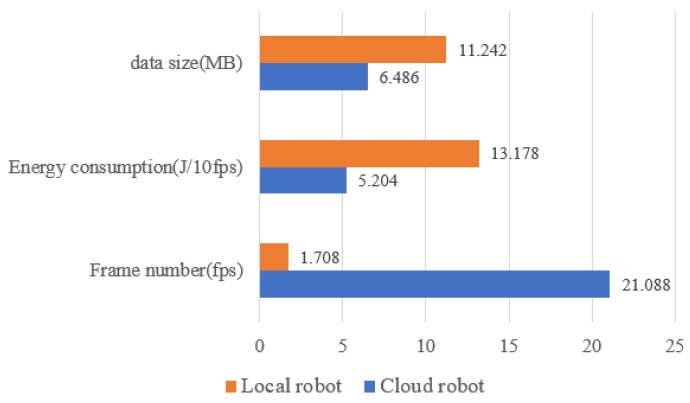
Performance comparison between different RGB-D SLAM.

According to the RGB-D SLAM algorithm, the performance analysis and comparison of the RGB-D SLAM with the cloud robot and the RGB-D SLAM with the local robot are described. In this paper, three indicators are used as the measurement criteria: (1) Frame number (Fps). Considering that for the same SLAM task, the number of images to be processed is certain, so the problem affecting the efficiency of SLAM is to calculate the speed of processing the image. (2) Energy consumption (J/10fps), the robot itself is resource-constrained and includes its energy. Therefore, the energy consumption caused by performing the same SLAM task is also an important indicator to measure the efficiency of SLAM, in order to accurately describe the energy consumption brought by SLAM, which measures the impact of the architecture, is measured by its average energy consumption per 10 frames. (3) Network circulation data (MB). The amount of data interaction between robots and the cloud, the speed of data interaction are also important indicators affecting SLAM. As shown in Table 6 and Figure 17, compared with the local robot’s RGB-D SLAM, the number of frames combined cloud robot is increased from 1.7fps to 21fps, and the energy consumption per 10 frames is reduced from 13.2J to 5.0J. From the comparison results of the frame number, the method combined with the cloud robot can process the collected data in time and get SLAM results faster. So, in practical application, the robot can obtain the map and location information which can be computed by the current sensing data acquisition in almost real-time. In terms of energy consumption, because the computer module of SLAM process which consumes more energy is put from the robot to the cloud for execution, the energy consumption of cloud-based robot method is significantly reduced, which means that the robot can accomplish more tasks with limited energy and reduce the frequency of the supply and the maintenance.

## 7. Conclusions

In this paper, the key aspects of the whole RGB-D SLAM algorithm are analyzed. The algorithm is improved from three aspects: feature extraction, point cloud stitching, and pose optimization. The combination of the RGB-D SLAM algorithm and cloud robot technology is realized, and the computationally intensive algorithm backend is offloaded to the cloud. The service architecture of SLAM combined with the cloud robot is designed by using the ROS software framework. The advantages of the algorithm are analyzed through experiments.

As a next step, the key issue we consider is how to realize data storage and sharing between multiple robots and cloud platforms when tasks need to be performed cooperatively in the process of combining cloud robotics and the RGB-D SLAM algorithm. We plan to design the detection module of the communication quality to determine whether the back-end function of RGB-D SLAM is executed in the cloud or locally.

## Figures and Tables

**Table 1:** The advantages and disadvantages of the red-green-blue-depth simultaneous localization and mapping (RGB-D SLAM) algorithm.

Advantages	Disadvantages
(1) No need to consider initial alignment like a monocular SLAM system	(1) RGB-D camera data contains noise and a lot of redundancy.
(2) No need to consume a lot of resources to compute depth like a binocular SLAM system	(2) The accuracy of feature matching and camera transformation matrix is not high
(3) RGB-D camera can provide rich color and depth images simultaneously	(3) Real-time performance is difficult to achieve in the construction of large-scale scene maps
(4) The construction of dense maps is relatively easy	
(5) It is helpful to realize the real-time 3D reconstruction system	

**Table 2:** Comparison of different platforms.

Parameter	C${}^{2}$TAM	Rapyuta	DAvinCi
Bandwidth	low	high	high
Latency	low	high	low
Power consumption	low	high	low

**Table 3:** Comparison of two registration methods

Registration Method	Running Time (ms)	Iterations	Average Error ($\times 10^{-6}$ m)
Classical ICP method	60,118	50	14.3
Improved SVD method	7183	10	8.8

**Table 4:** Comparison results of trajectory error indicators.

Index Terms	Method	
	Original RGB-D SLAM (m)	Improved RGB-D SLAM (m)
RMSE	0.095054	0.009679
Mean	0.093701	0.008139
Median	0.094659	0.006937
STD	0.016023	0.005229
MIN	0.047141	0.000787
MAX	0.145821	0.022873

**Table 5:** Time consumption comparison of two methods for map building.

Scene	Combined Cloud			Local
	Data Transfer Time (ms)	Execution Time (ms)	Time Consumption (ms)	Time Consumption (ms)
A	6546	4856	11,402	36,406
B	5009	4893	9902	35,782
C	6298	5421	11,719	41,010
D	5974	4782	10,756	39,546
E	7283	6438	13,721	42,162
Mean	6222	5278	11,500	38,981

**Table 6:** Performance comparison of two methods.

Scene	Combined Cloud			Local		
	Num. Frames (fps)	Energy Con. (J/10fps)	Data Size (MB)	Num. Frames (fps)	Energy Con. (J/10fps)	Data Size (MB)
A	20.28	4.83	5.67	1.72	12.94	10.95
B	21.25	5.21	6.88	1.78	13.97	11.24
C	21.64	4.94	7.53	1.65	12.98	11.36
D	20.92	5.11	5.43	1.73	13.32	11.18
E	21.35	5.03	6.92	1.66	13.38	11.48
Mean	21.088	5.204	6.486	1.708	13.178	11.242
